# Triple-drug Therapy With Bevacizumab, Irinotecan, and Temozolomide Plus Tumor Treating Fields for Recurrent Glioblastoma: A Retrospective Study

**DOI:** 10.3389/fneur.2019.00042

**Published:** 2019-01-31

**Authors:** Guangrong Lu, Mayank Rao, Ping Zhu, Buqing Liang, Rasheda T. El-Nazer, Ekokobe Fonkem, Meenakshi B. Bhattacharjee, Jay-Jiguang Zhu

**Affiliations:** ^1^The Vivian L. Smith Department of Neurosurgery, The University of Texas Health Science Center at Houston (UTHealth), McGovern Medical School, Houston, TX, United States; ^2^Epidemiology, Human Genetics and Environmental Sciences, School of Public Health, UTHealth School of Public Health, Houston, TX, United States; ^3^Baylor Scott and White Health, Temple, TX, United States; ^4^Department of Pathology and Laboratory Medicine, The University of Texas Health Science Center at Houston (UTHealth), McGovern Medical School, Houston, TX, United States

**Keywords:** recurrent glioblastoma (rGBM), temozolomide, bevacizumab, irinotecan, tumor treating fields (TTFields, Optune), chemotherapy

## Abstract

Clinical studies treating pediatric and adult solid tumors, such as glioblastoma (GBM), with a triple-drug regimen of temozolomide (TMZ), bevacizumab (BEV), and irinotecan (IRI) [TBI] have demonstrated various efficacies, but with no unexpected toxicities. The TBI regimen has never been studied in recurrent GBM (rGBM) patients. In this retrospective study, we investigated the outcomes and side effects of rGBM patients who had received the TBI regimen. We identified 48 adult rGBM patients with a median age of 56 years (range: 26–76), who received Tumor Treating Fields (TTFields) treatment for 30 days or longer, and concurrent salvage chemotherapies. The patients were classified into two groups based on chemotherapies received: TBI with TTFields (TBI+T, *N* = 18) vs. bevacizumab (BEV)-based chemotherapies with TTFields (BBC+T, *N* = 30). BBC regimens were either BEV monotherapy, BEV+IRI or BEV+CCNU. Patients in TBI+T group received on average 19 cycles of TMZ, 26 and 21 times infusions with BEV and IRI, respectively. Median overall survival (OS) and progression-free survival (PFS) for rGBM (OS-R and PFS-R) patients who received TBI+T were 18.9 and 10.7 months, respectively. In comparison, patients who received BBC+T treatment had OS-R and PFS-R of 11.8 (*P* > 0.05) and 4.7 (*P* < 0.05) months, respectively. Although the median PFS results were significantly different by 1.5 months (6.6 vs. 5.1) between TBI+T and BBC+T groups, the median OS difference of 14.7 months (32.5 vs. 17.8) was more pronounced, *P* < 0.05. Patients tolerated TBI+T or BBC+T treatments well and there were no unexpected toxicities. The most common side effects from TBI+T treatment included grade III hypertension (38.9%) and leukopenia (22.2%). In conclusion, the TBI regimen might play a role in the improvement of PFS-R and OS-R among rGBM patients. Prospective studies with a larger sample size are warranted to study the efficacy and toxicity of TBI+T regimen for rGBM.

## Introduction

Glioblastoma (GBM) is the most common and devastating primary malignant brain tumor in adults (1, 2). The standard of care (SOC) for newly diagnosed GBM includes maximal safe tumor resection, 6 weeks of concurrent radiation with temozolomide (TMZ), followed by maintenance (or adjuvant) TMZ for a total of 6 to 12 cycles (1). Angiogenesis is a hallmark of GBM, and it is mediated in part by vascular endothelial growth factor (VEGF). The VEGF pathway can be inhibited by bevacizumab (BEV), a humanized monoclonal antibody binding specifically to circulating VEGF-A which received accelerated approval by the United States Food and Drug Administration (FDA) for the treatment of recurrent GBM (rGBM) in May 2009 based on results from two phase II trials (3, 4). However, administration of BEV along with TMZ as an adjuvant treatment for newly diagnosed GBM did not improve median overall survival (OS) when compared to patients that received TMZ monotherapy (5, 6). Nevertheless, BEV remains as an anti-angiogenesis therapy for rGBM in USA, and it is administered biweekly with or without irinotecan (IRI) (7) depending on treating physicians. Other therapies have been applied to rGBM, including second craniotomy with or without carmustine implantation (Gliadel wafers), salvage radiation, dose-dense TMZ, nitrosoureas, carboplatin, PCV [procarbazine, lomustine (CCNU) and vincristine], etoposide or IRI (8–10). TMZ is not commonly used for rGBM therapy (11). Optune™, a tumor treating fields (TTFields) medical device, is a novel therapeutic modality that was approved by the FDA for both recurrent and newly diagnosed GBM in April 2011 and October 2015, respectively. The EF-14 phase III clinical trial (NCT00916409) using TTFields along with maintenance TMZ prolonged median OS to 20.9 months, which was superior to a median OS of 16.0 months in the control group treated with TMZ monotherapy for newly diagnosed GBM (12). Based on an analysis of population-based databases, the median OS in the general GBM population is between 8 and 11 months (13).

GBM consists of a heterogeneous population of cells that are genetically unstable and highly infiltrative. GBM tissues demonstrate angiogenesis and have variable sensitivities or resistance to chemotherapy, radiation, and other therapeutic modalities (14). Combination chemotherapy is the strategy of using chemotherapy drugs concurrently to yield additive or synergistic inhibitory effects to glioma cell growth and make it difficult for cancer cells to develop drug resistance (15, 16). However, no chemotherapy combination has yet been shown to extend survival time than current SOC treatment for rGBM patients through phase III studies. Current rGBM treatments are mainly monotherapy of CCNU, or BEV-based chemotherapy (BBC) with or without TTFields. BBC regimens can be either BEV, BEV+IRI, or BEV+CCNU. Retreatment with TMZ for rGBM is also reported in literature, but it is not as commonly used as BBC. There have been clinical trials evaluating the efficacy of combinations of two chemotherapy drugs for rGBM. TMZ-containing or BEV-containing two-drug combinations did not show better outcomes than monotherapy with TMZ or BEV alone (11). A combination of BEV and CCNU did demonstrate a survival benefit in a phase II study (17); however, a recently-completed phase III study demonstrated that treatment with the combination of BEV and CCNU (*N* = 288) did not confer a survival advantage over treatment with CCNU monotherapy (*N* = 149) in rGBM patients (18). The triple-drug combination of TMZ, BEV and IRI (TBI) has been studied in 3 different clinical trials treating GBM: a phase I study of 41 unresectable GBM patients (NCT00979017) (19), a phase II study of 75 newly diagnosed GBM subjects (NCT00597402) (20), and a phase I pilot study including 12 high-grade glioma (HGG) subjects (NCT00890786) (21). These studies demonstrated that the TBI regimen was reasonably tolerated among GBM and HGG patients. Six other clinical studies (Supplement Table 1) also used the TBI regimen and reported no unexpected toxicities. However, the TBI regimen has not hitherto been studied in rGBM.

The TBI regimen has not been approved by FDA for rGBM. However, the FDA does not restrict physicians' practice for “off-label” use of approved medications or their combinations (22). Neuro-oncologists at the Mischer Neuroscience Institute (MNI)/UTHealth have been treating rGBM patients with normal bone marrow function with the TBI regimen since 2010. The treatment recommendation of the TBI regimen for rGBM patients is based on three factors: (1) rGBM has poorer prognosis and BEV monotherapy has only limited benefit; (2) there is no effective therapy after BEV failure; (3) TBI is reasonably tolerated for both children and adults patients as summarized in Supplement Table 1. The senior author had anecdotal experiences treating rGBM patients with TBI and had observed higher chances of long-term (>3 years) survival of those patients. Thereafter, TBI is offered to selected patients with rGBM based on the physician's best knowledge, experience and judgement with risks and benefits being well-communicated to prospective patients. After FDA approval of TTFields for rGBM in 2011, TBI with TTFields was recommended to patients with rGBM. Other patients received TTFields through their participation of EF-14 clinical trial (NCT00916409) for newly diagnosed GBM (12) and continue using the device through tumor progression. After 8 years of experience and accumulation of a cohort of patients who received TBI therapy with TTFields, we conducted a retrospective study analyzing the survival benefit and adverse events from two groups of patients (TBI+T and BBC+T) from January 1, 2011 to March 31, 2018, collaborating with the neuro-oncology colleagues at Baylor Scott and White Health (BSWH). Our primary goal was to see if there was a survival benefit and document any new and unexpected adverse events in the two patient groups. Our secondary goal was to analyze the occurrence of grade III and IV AEs associated with these combination therapies.

## Materials and Methods

### Patient Selection

**Figure 1:**
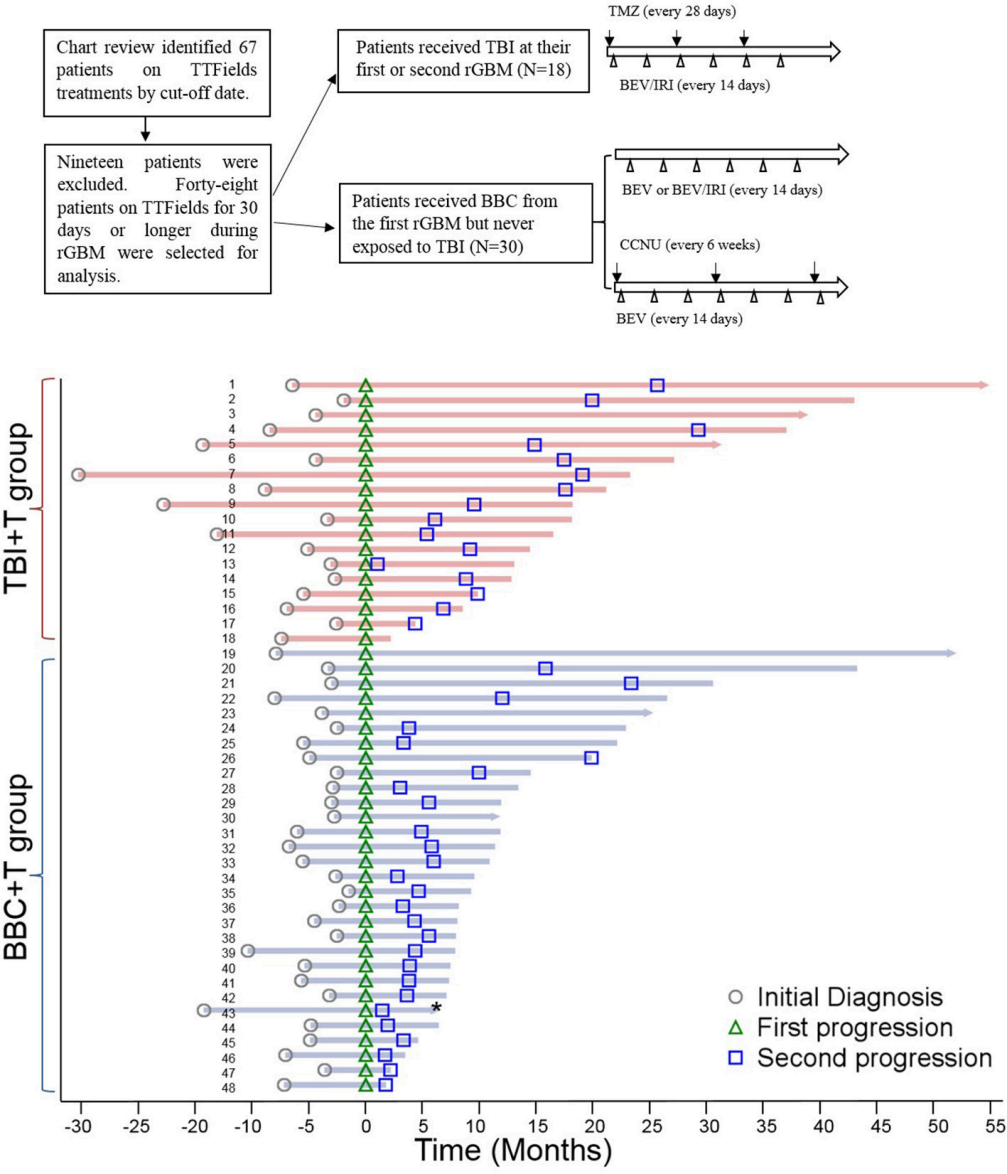
Data review flowchart and swimmer plot of patients who received TTFields for 30 days and longer. Data review flowchart showed the data selection process and treatments with different salvage chemotherapies in two subgroups. Patient's IDs are listed on each line within the swimmer plot. All patients are aligned according to the date of first disease progression (green triangle), and the second GBM progression dates are all marked (blue square). The horizontal bar with an arrow at the right end indicates subjects who were still alive as of 3/31/2018 while the blunt end bar indicates subjects who passed away. Survival status for patient number 43 in BBC+T group was unknown (*).

The study protocol was approved by the Committee for the Protection of Human Subjects at UTHealth and the Scott & White Institutional Review Board, respectively. Adult rGBM patients that received TTFields treatment for 30 days or longer starting January 1, 2011 were included in the study. Among 48 eligible patients, 20 of them started TTFields during the adjuvant chemotherapy phase and 28 subjects began TTFields during the salvage chemotherapy period. The subjects were further classified into two subgroups according to the chemotherapies received: TBI+T vs. BBC+T (Figure 1). The data collection cut-off date was March 31, 2018.

### Chemotherapies, TTFields and Adverse Events Grading

TTFields was delivered through 4 panel transducers on a shaved scalp according to the array map produced for each subject by NovoTAL™ (Novocure, Inc.) per the manufacturer and FDA guidelines. Transducers were replaced twice a week. The patients in the TBI+T group received concurrent TBI and TTFields at tumor recurrence. Daily TMZ (150 mg/M^2^) was given for the first 5 days of every 28-days cycle, BEV (10 mg/kg) and IRI (125 mg/M^2^) infusions were given on completion of at least one cycle. BEV plus IRI were administered every 2 weeks intravenously; dose adjustment, and/or treatment delay were allowed. No patient received enzyme inducing anti-epileptic medication. For the BBC+T group, the patients received BEV-based chemotherapy including BEV monotherapy or BEV-based two-drug combinations (BEV+IRI, or BEV+CCNU). Medical records and treatment histories were reviewed. Collected information included age, sex, body weight, diagnosis, surgery, lab results (complete blood count, comprehensive metabolic panel, liver and renal function tests, and urinalysis), MRI, pathology report, chemotherapy history, survival status, and adverse events (AE). Common Terminology Criteria for Adverse Events (CTCAE), version 4.0, was used to grade the severity of the AE.

### Statistical Analysis

The student's *t*-test or Pearson's χ^2^ test was applied to compare baseline characteristics of the groups. OS was calculated from the initial diagnosis of GBM to death, or the last follow up. PFS was calculated from the initial diagnosis of GBM to the date of first progression. OS-R was calculated from the date of diagnosis of first GBM progression to death or last follow-up in the majority of cases; in cases where TBI was started other than at first progression, it was calculated from corresponding progression date to death. PFS-R was calculated from the date of the first tumor progression to the second progression; in cases where TBI was started other than first progression, it was calculated from the corresponding progression date to the next progression. Patients who were alive or those whose survival status was unknown on March 31, 2018 were censored. The Kaplan-Meier method was applied to estimate OS, PFS, OS-R, and PFS-R. The difference in survival functions was tested by the generalized Wilcoxon and log-rank tests. A Swimmer-Plot was utilized to compare the time-lines of GBM history by treatment group. All statistical analyses were performed with SAS 9.4 (SAS Institute Inc., Cary, North Carolina). *P*-values were two-sided and considered statistically significant at *P* < 0.05.

## Results

### Patient Demographics and Treatments Received

A total of 48 GBM patients met the inclusion criteria (Figure 1). Forty patients were from MNI and eight patients were from BSWH. Twenty patients received TTFields after newly diagnosed GBM, with eight in the TBI+T group and twelve patients in the BBC+T group. The remaining 28 patients started TTFields therapy at GBM recurrence. As of March 31, 2018, 41 patients were deceased, six patients were alive, and the survival status of one patient was unknown. Median ages for the BBC+T and TBI+T groups were 58 (range: 26–76) and 53 (range: 29–70) years old, respectively. Patients received 6.1 ± 5.5 cycles of TMZ, 13.6 ± 12.3 BEV infusions, and 2.4 ± 6.7 IRI infusions in the BBC+T group; and 19.3 ± 13.3 cycles of TMZ, 25.9 ± 12.1 BEV infusions, and 21.3 ± 12.8 IRI infusions in the TBI+T group. The number of TMZ cycles included both adjuvant and salvage therapies. Details of the chemotherapies administered are presented in Table 1. Individual patient's disease status is presented in a swimmer plot in Figure 1.

**Table 1:** Baseline demographics, clinical treatment received and outcomes.

**Characteristics**	**BBC+T**	**TBI+T**
Total number of patients	30	18
Age (years, mean ± SD)	57.8 ± 11.6	52.3 ± 9.9
**SEX**, ***N*** **(%)**		
Male	19 (63.3%)	12 (66.7%)
Female	11 (36.7%)	6 (33.3%)
**SURGICAL INTERVENTION AFTER PROGRESSION**		
Stereotactic radiosurgery (SRS)	3	1
2nd or more surgical resection	5	2
Both craniotomy and SRS as indicated	5	4
TMZ (cycles)	6.1 ± 5.5^*^^#^	19.3 ± 13.3
BEV infusion (times)	13.6 ± 12.3	25.9 ± 12.1
BEV total amount (mg)	10,080 ± 9,622	23,115 ± 11,867
IRI infusion (times)	2.4 ± 6.7	21.3 ± 12.8
IRI total amount (mg)	552 ± 1,574	5,589 ± 3,486
**TTFIELDS THERAPY**		
Adjuvant user, *N* (%)	12 (40%)	8 (44.4%)
Salvage user, *N* (%)	18 (60%)	10 (55.6%)
Duration (days)	274 ± 309	534 ± 430
IDH1 mutant detected^**^	0/12	2/11
TERT promoter mutation detected^**^	6/12	1/11
EGFR mutant or amplification detected^**^	5/12	4/11
**OUTCOMES**		
OS in months, median (95%CI)	17.8 (13.3–19.9)	32.5 (17.0–49.0)^Ω^
PFS in months, median (95%CI)	5.1 (3.3–6.1)	6.6 (3.7–9.2)^Ω^
OS-R in months, median (95%CI)	11.8 (8.6–15.8)	18.9 (10.7–25.3)
PFS-R in months, median (95%CI)	4.7 (3.6–6.3)	10.7 (6.7–20.8)^Ω^

*SD, standard deviation; IDH, isocitrate dehydrogenase; TERT, telomerase reverse transcriptase; EGFR, epidermal growth factor receptor. In BBC+T group*,

* four patients never received maintenance TMZ due to severe myelosuppression during the 6-weeks CCRT period;

# *One patient received just 1 maintenance TMZ cycle before switching to another regimen that patient and his family had chosen*.

** *11 and 12 cases from TBI+T and BBC+T groups, respectively, had next generation sequencing reports from Foundation Medicine. CI, confidence interval*.

**Figure 2:**
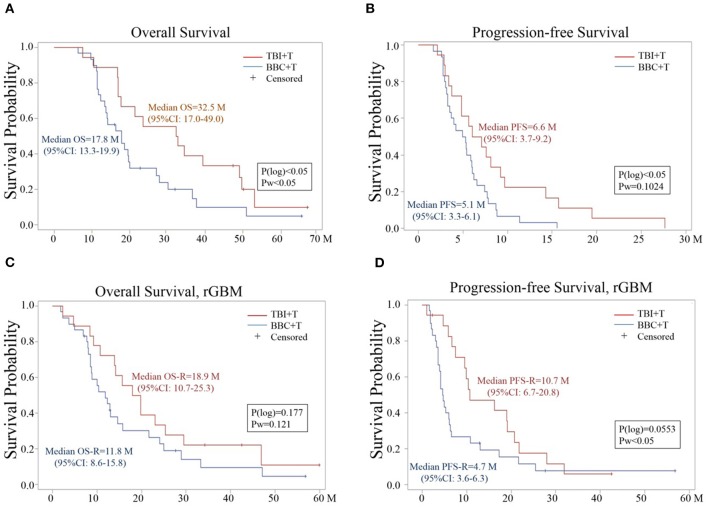
Kaplan –Meier Estimator analysis of **(A)** OS, **(B)** PFS, **(C)** OS-R, and **(D)** PFS-R for TBI+T and BBC+T groups. P(log) is a *p*-value based on log-rank test; Pw is a *p*-value based on Wilcoxon test. 95%CI: 95% confidence interval.

Ω *P < 0.05 in at least one of two types of statistical analysis (P-values are in Figure 2)*.

### Outcomes: Median OS, PFS, OS-R, and PFS-R Were Estimated by the Kaplan-Meier Method

As of March 31, 2018, three participants were alive in each of the two groups. Patients in the TBI+T group had median OS, PFS, OS-R and PFS-R of 32.5 (95%CI: 17.0–49.0), 6.6 (95%CI: 3.7–9.2), 18.9 (95%CI: 10.7–25.3), and 10.7 (95%CI: 6.7–20.8) months, respectively. In comparison, patients in the BBC+T group had median OS, PFS, OS-R and PFS-R of 17.8 (95%CI: 13.3–19.9), 5.1 (95%CI: 3.3–6.1), 11.8 (95%CI: 8.6–15.8), and 4.7 (95%CI: 3.6–6.3) months, respectively. When comparing treatment groups with statistical analysis, OS, PFS, and PFS-R values were significantly different with *P* < 0.05 from at least one type of analysis (Table 1 and Figure 2). OS-R value did not show a statistical difference between the two groups.

### Adverse Events

Grade III weight loss (23.3%) and hypertension (38.9%) were the most common side effects in BBC+T and TBI+T groups, respectively. Occurrences of grade III abnormal liver enzymes, proteinuria and hypertension were 10% or higher in the TBI+T than that in BBC+T groups. In contrast, occurrences of weight loss, and thrombocytopenia (grade III and IV combined) were more frequent in the BBC+T (23.3 and 26.7%) than the TBI+T (11.1 and 11.2%) group. Grade III venous thromboembolism (VTE, either pulmonary embolism or deep venous thrombosis) occurred in 6.7 and 11.1% among BBC+T and TBI+T groups, respectively. There was one case of perforated diverticulitis in the BBC+T group, which was likely related to BEV use in the setting of previous history of diverticulosis. TTFields use was associated with scalp erythema, erosion or ulceration. However, none of these AEs developed to grade III or IV level as listed in Table 2.

**Table 2:** Occurrence of grade III or higher adverse events during chemotherapy treatment.

	**BBC+T (*N* = 30)**	**TBI+T (*N* = 18)**
Adverse events (grade)	*N* (%)	*N* (%)
Anemia (III)	2 (6.7%)	0 (0%)
Cardiovascular system (III)	1 (3.3%)	1 (5.6%)
Edema (III)	1 (3.3%)	0 (0%)
GI perforation (III)	1 (3.3%)	0 (0%)
Hypertension (III)	4 (13.3%)	7 (38.9%)
Hypophosphatemia (III)	0 (0%)	1 (5.6%)
Leukopenia (III),	3 (10%)	4 (22.2%)
Leukopenia (IV)	3 (10%)	0 (0%)
Liver enzyme elevation (III)	1 (3.3%)	3 (16.7%)
Pneumonia (III)	1 (3.3%)	1 (5.6%)
Proteinuria (III)	1 (3.3%)	4 (22.2%)
Thrombocytopenia (III)	5 (16.7%)	1 (5.6%)
Thrombocytopenia (IV)	3 (10%)	1 (5.6%)
Venous thromboembolism^*^ (III)	2 (6.7%)	2 (11.1%)
Weight Loss (III)	7 (23.3%)	2 (11.1%)

* *VTE, pulmonary embolism and deep venous thrombosis combined*.

### One Example of Long PFS-R Period With TBI+T Treatment

**Figure 3:**
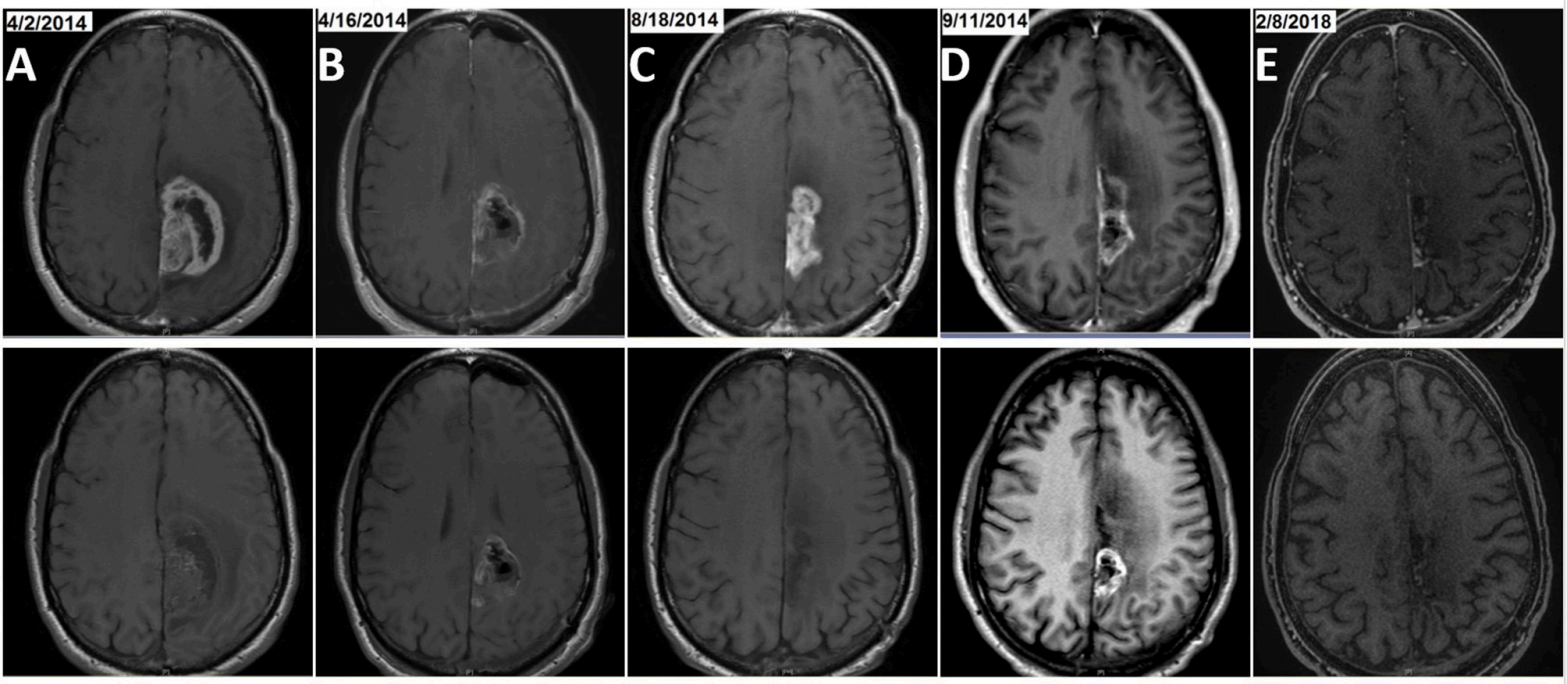
Example of MRI results from patient number 3, a long-term survivor in the TBI+T group. The patient was treated with initial craniotomy and concurrent radiation and temozolomide followed with maintenance TMZ. He was treated with TBI (22 treatments with BEV and IRI from March 2015 to January 2016) after his first GBM progression (rGBM) was resected. The patient finished a cumulative 30 cycles of TMZ when the TBI regimen was completed and he continued on TTFields treatment alone for 39 months as of March, 31, 2018. Two craniotomy surgeries were performed in April and September of 2014, respectively. Pre- and post-craniotomy MRIs, T1 with contrast for newly diagnosed GBM [**(A)**, 4/2/2014 and post-surgery at **(B)**, 4/16/2014] and rGBM [**(C)**, 8/18/2014 and post-surgery at **(D)**, 9/11/2014] as well as the most recent brain MRI immediately prior to the data cut-off date [**(E)**, 2/8/2018] are shown in upper row, with brain MRI T1 without contrast shown in the corresponding lower row.

Patient number 3 was first diagnosed with GBM in April, 2, 2014 when he was 52 years old. He was randomized to the SOC arm in the EF-14 clinical trial (NCT00916409). He chose to cross-over to the TTFields arm on January 22, 2015 with permission from the trial sponsor when interim data results were available. The patient completed 1 year treatment with the TBI regimen with TTFields after second craniotomy due to first tumor progression. There is no radiographic or clinical evidence of a second tumor progression during follow-up after completion of 1 year therapies with TBI treatment and TTFields as of March, 31 2018 (Figure 3). His GBM tumor tissue demonstrated O^6^-methylguanine-DNA methyltransferase (MGMT) promoter methylation, wild-type isocitrate dehydrogenase 1/2 (IDH1/2) and amplification of the epidermal growth factor receptor (EGFR) gene.

### One Example of Beneficial Use of TBI Regimen With SRS Gamma Knife at Second GBM Progression

**Figure 4:**
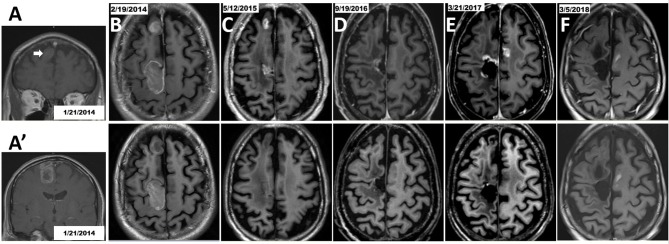
Therapy with TBI in combination with Gamma Knife stereotactic radiosurgery after the second GBM progression in patient number 5. Initial craniotomy was on 1/25/2014, but axial section images were not available in the pre-operative MRI. **(A)** Coronal view of anterior right frontal lesion (arrow). **(A')** Coronal view of posterior right frontal lesion. **(B)** Post-operative MRI. Tumor in the posterior right frontal lobe was removed while the mass at the right anterior frontal lobe was untouched. **(C)** MRI on 5/12/2015 showed that tumor in the right anterior frontal lobe progressed as a non-targeted lesion while on EF-14 clinical trial, and this lesion was treated with Gamma Knife on May 20, 2015. The patient had been treated with TBI therapy for 1 year and then it stopped as scheduled. **(D)** MRI on 9/19/2016 suggested tumor progression around the surgical cavity and he had a 2nd craniotomy on 9/25/2016. But pathology demonstrated no active tumor. This was not considered as progression of disease. **(E)** The 2nd disease progression was diagnosed based on MRI (3/21/2017) because of an appearance of a new lesion in the left hemisphere which was treated with Gamma Knife on 4/5/2017. The TBI regimen was restarted. **(F)** The progressive disease was well-controlled based on the latest MRI on 3/5/2018; lesions were treated with Gamma Knife followed by TBI regimen plus TTFields. Brain MRI T1 with contrast shown in upper row and brain MRI T1 without contrast shown in the corresponding lower row.

The TBI regimen was restarted after 1-year therapy for the first disease progression in patient number 5. This subject was a 56 years old male with an initial diagnosis of GBM on January 25, 2014 and he was enrolled in the EF-14 clinical trial. TBI was given as salvage chemotherapy and it was electively stopped after 1 year treatment as his GBM was stable. When his tumor showed second progression with a new lesion in his left frontal lobe, TBI was restarted in conjunction with stereotactic radiosurgery using the Gamma Knife. The patient tolerated the treatment very well, and MRI every 2 months demonstrated tumor shrinkage and his tumor has been stable as of March 31, 2018, 1 year after the second tumor progression (Figure 4). This patient completed 43 cycles TMZ and received 35 infusions of BEV/IRI and continues his full-time job as of March 31, 2018. His GBM tumor tissue showed MGMT promoter methylation. Molecular data on EGFR and IDH1/2 was not available.

## Discussion

This retrospective study demonstrated that TBI+T treatment (*N* = 18) for rGBM resulted in longer median OS and PFS-R than patients in the BBC+T group, *P* < 0.05. Although the median PFS was significantly different by 1.5 months (6.6 vs. 5.1), the difference of 14.7 months (32.5 vs. 17.8) in median OS was more pronounced. Because PFS-R values are also significantly prolonged by 6 months (10.7 vs. 4.7), it is possible that the TBI regimen better controlled tumor growth and played an important role in prolonging median OS. We believe that a lack of statistical significance of OS-R between TBI+T (*N* = 18) and BBC+T (*N* = 30) groups is probably due to the small sample sizes. Knowing that other factors, such as repeat surgery, Gamma Knife radiosurgery, molecular factors (MGMT promoter methylation status, and IDH1/2 mutation status) significantly affect prognosis, we cannot conclude with confidence that TBI alone or TBI+T are effective therapies for rGBM from this study. The TBI regimen produced moderate side effects including hypertension, leukopenia, and proteinuria. This study confirmed previous published results that TBI is a safe regimen with manageable and expected side effects (Supplement Table 1) (19–21, 23–27), which lends support to a proposed clinical trial testing this regimen in rGBM patients.

The goal of a combination chemotherapy is to achieve additive or synergistic effects in controlling aggressive GBM tumor cell growth. The rationale is to co-administer drugs that work by different molecular mechanisms. This has the potential to maximally reduce tumor cell growth and decrease the likelihood of drug resistance while simultaneously minimizing overlapping drug toxicity (16). TMZ causes methylation of DNA at the O^6^ and N^7^ positions of guanine, and it is excreted mainly by the kidney, and commonly induces myelosuppression (1, 28). BEV blocks angiogenesis by neutralizing VEGF, and it is metabolized and eliminated via the reticuloendothelial system. BEV causes hypertension, proteinuria, and it interferes with wound healing (3, 4). SN-38, an active metabolite of IRI, stabilizes the topoisomerase I-DNA complex, and it prevents DNA single-strand breaks from being re-ligated during DNA replication, which could lead to double-strand breaks and ultimately cause cell death (29, 30). Fecal excretion is the major elimination pathway for IRI, and it commonly causes diarrhea and myelosuppression (31). These known side effects are reversible after discontinuing the offending drug. Treatment delay or holding one or more chemotherapy drug(s) is also allowed if myelosuppression is identified prior to each cycle of drug therapy. TTFields are a regional physical treatment, which disrupts mitosis of proliferating cells. The transducer arrays that deliver TTFields cause scalp lesions, which can be managed by shifting the array positions on the scalp during array changes. There were no reports of systemic side effects from TTFields when combined with TMZ (32), neither was there evidence that continuous use of TTFields adversely affected quality of life, cognition, or functional status (33, 34).

When compared to the BBC+T group, the TBI+T group was more frequently associated with grade III liver enzyme elevation, hypertension and proteinuria. BEV caused 13.3 and 38.9% of hypertension in the BBC+T and TBI+T groups, respectively. Hypertension can be managed with anti-hypertensive medications or discontinuation of BEV. Elevation of liver enzymes may be attributed to TMZ; its occurrence in the TBI+T group (16.7%) is higher than that in the BBC+T group (3.3%). Myelosuppression (leukopenia and thrombocytopenia) is another side effect from both TMZ and IRI that requires close monitoring. Leukopenia toxicity from TMZ and/or IRI was usually reversible without medical treatment. However, some patients needed granulocyte colony-stimulating factor for an accelerated recovery. Patients who develop myelosuppression from radiation and/or TMZ regimens, including TBI regimen, have a high risk for developing pneumocystis jirovecii *pneumonia* (PcP), which can be deadly (35). Thus it is very important to start PcP prophylaxis to avoid opportunistic infections in patients with evidence of lymphopenia. For those patients who experienced moderate thrombocytopenia and liver enzyme elevation, it usually took 1–2 weeks to recover, and resuming treatment with TMZ was feasible. When grade III or IV myelosuppression events occur during the initial 6-weeks concurrent chemo-radiation therapy (CCRT) period, TMZ was discontinued permanently. Three patients required platelet transfusion because grade IV thrombocytopenia occurred during CCRT, and they were treated with BEV monotherapy during the first GBM progression because BEV is not known to cause myelosuppression. TMZ-induced severe myelosuppression is relatively rare and it happens mainly during CCRT (36). In line with this, the BBC+T group in our study had a higher incidence (26.7%) of thrombocytopenia than the TBI+T group (11.2%), grades III and IV combined (Table 2). There was one case of perforated diverticulitis in the BBC+T group, which was likely related to BEV use with the patient's history of diverticulosis. This is a known severe side effect from BEV use (37).

VTE was treated with low molecular weight heparin (1 mg/kg subcutaneous injection, twice a day for 3 months), and some patients had an inferior vena cava filter placement in addition to low molecular weight heparin therapy. Nabi et al. reported that administration of BEV was related to a higher incidence of VTE in GBM patients (38). However, it is difficult to establish a cause-effect relationship of VTE among patients receiving the TBI chemotherapy regimen because GBM patients' hypercoagulable state itself can lead to VTE (39, 40). In addition, the incidence of VTE increases as patients live longer. We believe that the increased rate of VTE in our study is likely due to a combination of the hypercoagulable state of GBM and BEV application. We did not observe any hemorrhage or other bleeding events while patients on BEV received therapeutic low molecular weight heparin.

A post-mortem study on organ toxicity after long-term use of TBI is ongoing at MNI/UTHealth. This study enrolled 66 decedents as of March 31, 2018. Autopsy reports from 24 decedents who received TBI treatments demonstrate no unexpected permanent organ toxicity/injury on gross and microscopic examination (unpublished data) (41). Three studies demonstrated that the TBI regimen was tolerable among GBM patients without unexpected toxicities (7, 19–21). The TBI regimen was also well-tolerated among pediatric patients with medulloblastoma (42) and neuroblastoma (24). A dramatic response from TBI regimen therapy was reported in a patient diagnosed with recurrent medulloblastoma with widespread osseous metastases (23). Although these studies have small sample sizes, they have all demonstrated that the TBI regimen is safe for patient use.

Guidance regarding the use of TMZ for newly diagnosed GBM includes whether GBM tissue harbors a methylated MGMT promoter, but there is no consensus on treating rGBM with TMZ alone or a TMZ-containing regimen. Research reports that the MGMT promoter within tumor tissue can convert from un-methylated to methylated status between initial and recurrent GBM, and *vice versa* (43). Thus, not treating rGBM patients with TMZ may not be justified, or at least the tumor MGMT promoter methylation status requires re-evaluation before making a therapeutic decision. Individual cases of as many as 101 (44) and 108 (45) cycles of TMZ including its use for recurrent glioma, did not report unexpected toxicities and treatment was effective in controlling tumor regrowth. Perry et al. demonstrated that re-challenging rGBM patients with continuous, dose-intense TMZ at 50 mg/M^2^/d was effective, especially among patients whose tumor progressed during the first six cycles of conventional adjuvant TMZ therapy or after a treatment-free interval (46). In our study, restarting the TBI regimen including TMZ as a component for patient number 5 elicited good disease control for his second GBM progression (Figure 4). Aguilera *et al* reported a 55-months treatment with TBI to a child diagnosed with relapsed medulloblastoma; the patient tolerated the treatment well and remained progression-free of the disease (42). The TBI regimen has been tested in several clinical trials treating pediatric and adolescent solid tumors (NCT00993044 (25), NCT02308527, NCT01114555 (24), NCT0121437 (26), NCT00876993, and NCT0118964327) (Supplement Table 1). The most recent study concluded that adding BEV to TMZ/IRI did not improve response rate compared to the TMZ/IRI combination in treating refractory or relapsed neuroblastoma (24).

When comparing a phase II clinical trial with TBI (20) to two large-scale phase III clinical trials (5, 6) in which the TMZ/BEV combination was used as adjuvant therapy for newly diagnosed GBM (Supplement Table 2), we noticed that IRI was the only component that differed in the adjuvant therapy phase. In the study using TBI as adjuvant therapy (*N* = 75), the median OS and PFS were 21.2 months (95% CI: 17.2–25.4) and 14.2 months (95% CI: 12–16), respectively (20). In comparison, studies of TMZ/BEV as adjuvant therapy elicited median OS and PFS about 16.5 and 10.6 months, respectively (5, 6). The adjuvant TBI regimen elicited better median OS and PFS outcomes when compared to two major phase III TMZ/BEV clinical trials. However, the research focus of the neuro-oncology community shifted to BEV later and no further studies were performed on the TBI regimen in the GBM population. We do not believe that IRI has a significant impact as a component in a two-drug combination regimen because BEV/IRI and TMZ/IRI combinations have been studied repeatedly and there has been no OS-R benefit (11, 47, 48). There has been no substantial progress in the development of novel chemotherapy drugs for rGBM and clinicians are limited to a few chemotherapy choices. Therefore, we believe that prospective studies using a combination of traditional chemotherapy drugs such as TMZ and IRI should be evaluated in combination with BEV and TTFields.

There are inherent limitations to our study because of its retrospective nature. Calculation of OS, PFS OS-R and PFS-R in this study is different from published data in clinical trials because clinical trials calculate survival from the day of randomization. In contrast, retrospective studies and population data analysis calculate survival day from the date of diagnosis. The execution of the treatment plan, especially when more treatments were combined, can be affected by insurance coverage. Some patients tended not to choose the TTFields device due to cosmetic concerns. Other limitations of our study include the small sample size, difficulty to control bias such as robust bone marrow functions, and missing data on MGMT promoter methylation and specific genetic tests. However, the decision to treat patients with TBI is not limited by molecular test results or Karnofsky performance score. More patients may potentially benefit from the combination treatment as long as their bone marrow functions are acceptable.

## Conclusion

Our retrospective study demonstrated that the TBI chemotherapy regimen with TTFields treatment has the potential to prolong median OS and PFS-R for rGBM patients. This regimen caused moderate, but manageable side effects without unexpected severe toxicities. There has been no novel effective treatment for rGBM in nearly a decade and the toxicity of the TBI regimen has been well-documented from its limited use among GBM and other brain tumor populations including pediatric patients. We strongly believe that a prospective clinical trial using TBI to treat rGBM with or without TTFields is warranted to investigate its efficacy and toxicity.

## Informed Consent

The Title of this approved project by the Committee for the Protection of Human Subjects at UTHealth was “*Chart review of outcome of GBM patients who received triple chemotherapies for progressive disease*.” No consent form is required for patients to sign for participation of the study.

## Author Contributions

GL: regulatory paperwork application to IRB, extract raw data, data management and analysis, draft manuscript. MR: review medical record and data extraction at UTHealth site, read, and edit manuscript. PZ: data analysis-Swimmer plot, K-M curves, Log-rank, and Wilcoxon tests, read, and edit manuscript. BL: regulatory paperwork to BSWH IRB, review medical record and data extraction at BSWH site, read, and edit manuscript. RE-N: review medical record and data extraction at BSWH site, read, and edit manuscript. EF: sub-I at BSWH site, treat patients with SOC, obtained IRB approval, pull patients' list, review cases for data extraction, read, and edit manuscript. MB: review histology slides and confirm pathological diagnosis of rGBM, read, and edit manuscript. J-JZ: senior author, treated patient with triple chemotherapy, manage side effects, original concept provider, obtained IRB approval, collaborate with Sub-I, read and revise manuscript, and obtain fund to support this study.

### Conflict of Interest Statement

EF reports receiving commercial research grants from Novocure, Abbvie, and Nativis. J-JZ reports receiving commercial research grants from NRG Oncology and Radiation Therapy Oncology Group (RTOG) Foundation, Boston Biomedical, Sumitomo Dainippon Pharma Global Oncology, DEKK-TEC, Inc., Diffusion Pharmaceuticals LLC., Five Prime Therapeutics, Inc., Immuno-Cellular Therapeutics LTD., Novocure, Inc, and Tocagen, Inc. The remaining authors declare that the research was conducted in the absence of any commercial or financial relationships that could be construed as a potential conflict of interest.

## References

[1] Stupp R Mason WP van den Bent MJ Weller M Fisher B Taphoorn MJ. Radiotherapy plus concomitant and adjuvant temozolomide for glioblastoma. N Engl J Med. (2005) 352:987–96. 10.1056/NEJMoa043330 15758009

[2] Huang RY Neagu MR Reardon DA Wen PY. Pitfalls in the neuroimaging of glioblastoma in the era of antiangiogenic and immuno/targeted therapy - detecting illusive disease, defining response. Front Neurol. (2015) 6:33. 10.3389/fneur.2015.00033 25755649 PMC4337341

[3] Friedman HS Prados MD Wen PY Mikkelsen T Schiff D Abrey LE. Bevacizumab alone and in combination with irinotecan in recurrent glioblastoma. J Clin Oncol. (2009) 27:4733–40. 10.1200/JCO.2008.19.8721 19720927

[4] Kreisl TN Kim L Moore K Duic P Royce C Stroud I. Phase II trial of single-agent bevacizumab followed by bevacizumab plus irinotecan at tumor progression in recurrent glioblastoma. J Clin Oncol. (2009) 27:740–5. 10.1200/JCO.2008.16.3055 19114704 PMC2645088

[5] Gilbert MR Dignam JJ Armstrong TS Wefel JS Blumenthal DT Vogelbaum MA. A randomized trial of bevacizumab for newly diagnosed glioblastoma. N Engl J Med. (2014) 370:699–08. 10.1056/NEJMoa1308573 24552317 PMC4201043

[6] Chinot OL Wick W Mason W Henriksson R Saran F Nishikawa R. Bevacizumab plus radiotherapy-temozolomide for newly diagnosed glioblastoma. N Engl J Med. (2014) 370:709–22. 10.1056/NEJMoa1308345 24552318

[7] Vredenburgh JJ Desjardins A Reardon DA Friedman HS. Experience with irinotecan for the treatment of malignant glioma. Neuro Oncol. (2009) 11:80–91. 10.1215/15228517-2008-075 18784279 PMC2718962

[8] Brem H Piantadosi S Burger PC Walker M Selker R Vick NA. Placebo-controlled trial of safety and efficacy of intraoperative controlled delivery by biodegradable polymers of chemotherapy for recurrent gliomas. The polymer-brain tumor treatment group. Lancet (1995) 345:1008–12. 10.1016/S0140-6736(95)90755-6 7723496

[9] Butowski NA Sneed PK Chang SM. Diagnosis and treatment of recurrent high-grade astrocytoma. J Clin Oncol. (2006) 24:1273–80. 10.1200/JCO.2005.04.7522 16525182

[10] Yung WK Albright RE Olson J Fredericks R Fink K Prados MD. A phase II study of temozolomide vs. procarbazine in patients with glioblastoma multiforme at first relapse. Br J Cancer (2000) 83:588–93. 10.1054/bjoc.2000.1316 10944597 PMC2363506

[11] Weller M Cloughesy T Perry JR Wick W. Standards of care for treatment of recurrent glioblastoma–are we there yet? Neuro Oncol. (2013) 15:4–27. 10.1093/neuonc/nos273 23136223 PMC3534423

[12] Stupp R Taillibert S Kanner A Read W Steinberg D Lhermitte B. Effect of tumor-treating fields plus maintenance temozolomide vs maintenance temozolomide alone on survival in patients with glioblastoma: a randomized clinical trial. JAMA (2017) 318:2306–16. 10.1001/jama.2017.18718 29260225 PMC5820703

[13] Zhu P Du XL Lu G Zhu JJ. Survival benefit of glioblastoma patients after FDA approval of temozolomide concomitant with radiation and bevacizumab: a population-based study. Oncotarget (2017) 8:44015–31. 10.18632/oncotarget.17054 28467795 PMC5546458

[14] Soeda A Hara A Kunisada T Yoshimura S Iwama T Park DM The evidence of glioblastoma heterogeneity. Sci Rep. (2015) 5:7979 10.1038/srep07979 25623281 PMC4306917

[15] Frei E Karon M Levin RH Freireich EJ Taylor RJ Hananian J. The effectiveness of combinations of antileukemic agents in inducing and maintaining remission in children with acute leukemia. Blood (1965) 26:642–56. 5321112

[16] Al-Lazikani B Banerji U Workman P. Combinatorial drug therapy for cancer in the post-genomic era. Nat Biotechnol. (2012) 30:679–92. 10.1038/nbt.2284 22781697

[17] Taal W Oosterkamp HM Walenkamp AM Dubbink HJ Beerepoot LV Hanse MC. Single-agent bevacizumab or lomustine versus a combination of bevacizumab plus lomustine in patients with recurrent glioblastoma (BELOB trial): a randomised controlled phase 2 trial. Lancet Oncol. (2014) 15:943–53. 10.1016/S1470-2045(14)70314-6 25035291

[18] Wick W Gorlia T Bendszus M Taphoorn M Sahm F Harting I. Lomustine and bevacizumab in progressive glioblastoma. N Engl J Med. (2017) 377:1954–63. 10.1056/NEJMoa1707358 29141164

[19] Peters KB Lou E Desjardins A Reardon DA Lipp ES Miller E. Phase II trial of upfront bevacizumab, irinotecan, and temozolomide for unresectable glioblastoma. Oncologist (2015) 20:727–8. 10.1634/theoncologist.2015-0135 26025933 PMC4492239

[20] Vredenburgh JJ Desjardins A Reardon DA Peters KB Herndon JE Marcello J. The addition of bevacizumab to standard radiation therapy and temozolomide followed by bevacizumab, temozolomide, and irinotecan for newly diagnosed glioblastoma. Clin Cancer Res. (2011) 17:4119–24. 10.1158/1078-0432.CCR-11-0120 21531816 PMC3117928

[21] Hummel TR Salloum R Drissi R Kumar S Sobo M Goldman S. A pilot study of bevacizumab-based therapy in patients with newly diagnosed high-grade gliomas and diffuse intrinsic pontine gliomas. J Neurooncol. (2016) 127:53–61. 10.1007/s11060-015-2008-6 26626490

[22] Gupta SK Nayak RP. Off-label use of medicine: perspective of physicians, patients, pharmaceutical companies and regulatory authorities. J Pharmacol Pharmacother. (2014) 5:88–92. 10.4103/0976-500X.130046 24799811 PMC4008928

[23] Bonney PA Santucci JA Maurer AJ Sughrue ME McNall-Knapp RY Battiste JD. Dramatic response to temozolomide, irinotecan, and bevacizumab for recurrent medulloblastoma with widespread osseous metastases. J Clin Neurosci. (2016) 26:161–3. 10.1016/j.jocn.2015.10.022 26777082

[24] Modak S Kushner BH Basu E Roberts SS Cheung NV. Combination of bevacizumab, irinotecan, and temozolomide for refractory or relapsed neuroblastoma: results of a phase II study. Pediatr Blood Cancer (2017) 64:e26448. 10.1002/pbc.26448 28111925 PMC5555116

[25] Venkatramani R Malogolowkin M Davidson TB May W Sposto R Mascarenhas L. A phase I study of vincristine, irinotecan, temozolomide and bevacizumab (vitb) in pediatric patients with relapsed solid tumors. PLoS ONE (2013) 8:e68416. 10.1371/journal.pone.0068416 23894304 PMC3718768

[26] Levy A Krailo M Chi S Williams-Hughes C Bancroft M Villaluna D Pdct-09. temozolomide with irinotecan versus temozolomide, irinotecan plus bevacizumab for recurrent medulloblastoma/cns pnet of childhood: report of a cog randomized phase ii screening trial. Neuro-Oncology (2017) 19(suppl_6):vi186 10.1093/neuonc/nox168.753 PMC8764558 33844469

[27] Magnan HD. Price A. Chou AJ Riedel E Wexler LH Ambati SR A pilot trial of irinotecan, temozolomide and bevacizumab (ITB) for treatment of newly diagnosed patients with desmoplastic small round cell tumor (DSRCT). J Clin Oncol. (2017) 35(15_suppl.):11050 10.1200/JCO.2017.35.15_suppl.11050

[28] Patel M McCully C Godwin K Balis FM. Plasma and cerebrospinal fluid pharmacokinetics of intravenous temozolomide in non-human primates. J Neurooncol. (2003) 61:203–7. 10.1023/A:1022592913323 12675312

[29] Gilbert DC Chalmers AJ El-Khamisy SF. Topoisomerase I inhibition in colorectal cancer: biomarkers and therapeutic targets. Br J Cancer (2012) 106:18–24. 10.1038/bjc.2011.498 22108516 PMC3251848

[30] Hsiang YH Hertzberg R Hecht S Liu LF. Camptothecin induces protein-linked DNA breaks via mammalian DNA topoisomerase I. J Biol Chem. (1985) 260:14873–8. 2997227

[31] Slatter JG Schaaf LJ Sams JP Feenstra KL Johnson MG Bombardt PA. Pharmacokinetics, metabolism, and excretion of irinotecan (CPT-11) following I.V. infusion of [(14)C]CPT-11 in cancer patients. Drug Metab Dispos. (2000) 28:423–33. 10725311

[32] Stupp R Taillibert S Kanner AA Kesari S Steinberg DM Toms SA. Maintenance therapy with tumor-treating fields plus temozolomide vs. temozolomide alone for glioblastoma: a randomized clinical trial. JAMA (2015) 314:2535–43. 10.1001/jama.2015.16669 26670971

[33] Zhu JJ Demireva P Kanner AA Pannullo S Mehdorn M Avgeropoulos N. Health-related quality of life, cognitive screening, and functional status in a randomized phase III trial (EF-14) of tumor treating fields with temozolomide compared to temozolomide alone in newly diagnosed glioblastoma. J Neurooncol. (2017) 135:545–52. 10.1007/s11060-017-2601-y 28849310 PMC5700237

[34] Stupp R Wong ET Kanner AA Steinberg D Engelhard H Heidecke V. NovoTTF-100A versus physician's choice chemotherapy in recurrent glioblastoma: a randomised phase III trial of a novel treatment modality. Eur J Cancer (2012) 48:2192–202. 10.1016/j.ejca.2012.04.011 22608262

[35] De Vos FY Gijtenbeek JM Bleeker-Rovers CP van Herpen CM. Pneumocystis jirovecii pneumonia prophylaxis during temozolomide treatment for high-grade gliomas. Crit Rev Oncol Hematol. (2013) 85:373–82. 10.1016/j.critrevonc.2012.08.002 22925496

[36] Kourelis TV Buckner JC Gangat N Patnaik MM. Temozolomide induced bone marrow suppression–A single institution outcome analysis and review of the literature. Am J Hematol. (2015) 90:E183–4. 10.1002/ajh.24066 26010271

[37] Hapani S Chu D Wu S. Risk of gastrointestinal perforation in patients with cancer treated with bevacizumab: a meta-analysis. Lancet Oncol. (2009) 10:559–68. 10.1016/S1470-2045(09)70112-3 19482548

[38] Nabi S Kahlon P Bozorgnia F Arshad A Mikkelsen T Donthireddy V. Predictors of venous thromboembolism in patients with glioblastoma. Pathol Oncol Res. (2016) 22:311–6. 10.1007/s12253-015-0008-7 26547860

[39] Edwin NC Khoury MN Sohal D McCrae KR Ahluwalia MS Khorana AA. Recurrent venous thromboembolism in glioblastoma. Thromb Res. (2016) 137:184–8. 10.1016/j.thromres.2015.11.027 26657302

[40] Semrad TJ O'Donnell R Wun T Chew H Harvey D Zhou H. Epidemiology of venous thromboembolism in 9489 patients with malignant glioma. J Neurosurg. (2007) 106:601–608. 10.3171/jns.2007.106.4.601 17432710

[41] Lu G Rao M Zhu P Tian X Linendoll N Pilichowska N Postmortem evaluation of end-organ toxicity in patients with glioblastoma treated with temozolomide, bevacizumab and irinotecan. Neuro-oncology (2017) 19((suppl_3)):iii 10.1200/jco.2011.29.15_suppl.e19579 36203027

[42] Aguilera D Mazewski C Fangusaro J MacDonald TJ McNall-Knapp RY Hayes LL. Response to bevacizumab, irinotecan, and temozolomide in children with relapsed medulloblastoma: a multi-institutional experience. Childs Nerv Syst. (2013) 29:589–96. 10.1007/s00381-012-2013-4 23296323 PMC3963487

[43] O'Regan CJ Kearney H Beausang A Farrell MA Brett FM Cryan JB. Temporal stability of MGMT promoter methylation in glioblastoma patients undergoing STUPP protocol. J Neurooncol. (2017) 137:233–40. 10.1007/s11060-017-2722-3 29264834

[44] Barbagallo GM Paratore S Caltabiano R Palmucci S Parra HS Privitera G. Long-term therapy with temozolomide is a feasible option for newly diagnosed glioblastoma: a single-institution experience with as many as 101 temozolomide cycles. Neurosurg Focus (2014) 37:E4. 10.3171/2014.9.FOCUS14502 25434389

[45] Hirano H Kawahara T Niiro M Yonezawa H Takajyou T Ohi Y Anaplastic astrocytoma cells not detectable on autopsy following long-term temozolomide treatment: a case report. Mol Clin Oncol. (2017) 6:321–6. 10.3892/mco.2017.1160 28451406 PMC5403526

[46] Perry JR Bélanger K Mason WP Fulton D Kavan P Easaw J. Phase II trial of continuous dose-intense temozolomide in recurrent malignant glioma: rescue study. J Clin Oncol. (2010) 28:2051–7. 10.1200/JCO.2009.26.5520 20308655

[47] Quinn JA Jiang SX Reardon DA Desjardins A Vredenburgh JJ Friedman AH Phase II trial of temozolomide (TMZ) plus irinotecan (CPT-11) in adults with newly diagnosed glioblastoma multiforme before radiotherapy. J Neurooncol. (2009) 95:393–400. 10.1007/s11060-009-9937-x 19533023 PMC2835159

[48] Fountzilas G Karkavelas G Kalogera-Fountzila A Karina M Ignatiadis M Koukoulis G. Post-operative combined radiation and chemotherapy with temozolomide and irinotecan in patients with high-grade astrocytic tumors. A phase II study with biomarker evaluation. Anticancer Res. (2006) 26:4675–86. 17214326

