# Supplementary material for: Triple-drug Therapy With Bevacizumab, Irinotecan, and Temozolomide Plus Tumor Treating Fields for Recurrent Glioblastoma: A Retrospective Study
Source: Front Neurol. 2019 Jan 31;10:42. doi: 10.3389/fneur.2019.00042 (PMC6366009; doi:10.3389/fneur.2019.00042)
Supplement: Supplementary file 1 [file Data_Sheet_1.docx]

Supplement Table 1. Summary of clinical studies of TBI regimen for different solid tumors

| NCT number | Study arms | Study phase | Targeted Disease | Current status | Major Conclusions |
| --- | --- | --- | --- | --- | --- |
| ***Pediatric and adolescent CNS tumor*** | | | | | |
| 02308527 | 6 | II | neuroblastoma (NB) | Active enrolling |  |
| 00876993 | 5 | I | Refractory CNS Tumors | Complete |  |
| 01217437^28^ | 2 | II | Recurrent or Refractory MB or CNS PNET. | Active, but not enrolling | 1). TBI was relatively well tolerated in heavily pre-treated cohort and warrant further investigation. 2). TBI improved both EFS and OS by 3 months in children with recurrent disease. ^28^ |
| ***Pediatric and Adolescent solid tumors*** | | | | | |
| 00993044^27^ | 1 | I | Recurrent solid tumors, non-hemato-poietic origin. | Complete | 1). Encouraging anti-tumor activity was noted. 2). Irinotecan 50 mg/M^2^ /day for 5 days was the MTD when combined with vincristine, TMZ and BEV administered on a 21 day schedule.^27^ |
| 01189643^29^ | 2 | I | Newly Diagnosed DSRCT | Active, but not enrolling | 1). TBI is active in patients with Desmoplastic Small Round Cell Tumor (DSRCT). 2). It is feasible to combine these agents with standard chemotherapy without greater than expected toxicity. |
| ***Children and Young Adult Brain Tumors*** | | | | | |
| 00890786^21^ | 2 | I | High grade glioma and DIPG | Complete | TBI regimen is feasible and tolerable in newly diagnosed children and young adults with HGG and diffuse intrinsic pontine glioma (DIPG).^21^ |
| 01114555^26^ | 1 | II | Refractory or relapsed NB | Complete | 1). TBI was well tolerated. 2). Adding BEV did not improve response rates in resistant NB compared to historical data. |
| ***Adult GBM*** | | | | | |
| 00597402^20^ | 1 (N=75) | II | Newly diagnosed GBM | Complete | 1). Adding BEV to standard radiation therapy and TMZ, followed by TBI, for the treatment of newly diagnosed GBM has moderate toxicity and may improve efficacy compared with historical controls. 2). The median OS was 21.2 months.^20^ |
| 00979017^19^ | 1 (N=41) | I | Unresectable GBM | Complete | Treatment with TBI is tolerable and can lead to radiographic response in unresectable and/or sub-totally resected GBM. |
