# Supplementary material for: Triple-drug Therapy With Bevacizumab, Irinotecan, and Temozolomide Plus Tumor Treating Fields for Recurrent Glioblastoma: A Retrospective Study
Source: Front Neurol. 2019 Jan 31;10:42. doi: 10.3389/fneur.2019.00042 (PMC6366009; doi:10.3389/fneur.2019.00042)
Supplement: Supplementary file 2 [file Data_Sheet_2.docx]

Supplement Table 2. Compare clinical trial outcomes between one TBI protocol and two TMZ/BEV combinations treating newly diagnosed GBM as adjuvant therapy.

| Clinical trial | Phase | Adjuvant chemotherapy | Subjects (n) | | OS (months) | | PFS (months) | |
| --- | --- | --- | --- | --- | --- | --- | --- | --- |
|  |  |  | Control arm | Study arm | Control arm | Study arm | Control arm | Study arm |
| NCT00597402^20^ | II | TMZ/BEV/IRI |  | 75 | NA | 21.2 | NA | 14.2 |
| NCT00884741^5^ | III | TMZ/BEV |  | 312 |  | 15.7* |  | 10.7** |
|  |  | TMZ | 309 |  | 16.1* |  | 7.3** |  |
| [NCT00943826](http://clinicaltrials.gov/show/NCT00943826)^6^ | III | TMZ/BEV |  | 458 |  | 16.8^#^ |  | 10.6^##^ |
|  |  | TMZ | 463 |  | 16.7^#^ |  | 6.2^##^ |  |

* and ^#^ indicate non-statistical significance between study arm and control arm; ** and ^##^ indicate statistical significant difference between study arm and control arm.
